# The human lung microbiome progressively diminishes in the distal alveolar regions

**DOI:** 10.1038/s41522-026-01047-y

**Published:** 2026-06-15

**Authors:** Pauline Potratz, Antje Häder, Laura Kursawe, Judith Kikhney, Nikolaus Gaßler, Thurid Lauf, Lukáš Radosa, Tim Sandhaus, Patrick von Samson, Xiuqiang Chen, Lu Wang, Axel A. Brakhage, Annette Moter, Gianni Panagiotou, Torsten Doenst, Stefanie Deinhardt-Emmer, Bettina Löffler

**Affiliations:** 1 Institute of Medical Microbiology, Jena University Hospital, Jena, Germany; 2 University of Leipzig Medical Center, Institute of Medical Microbiology and Virology, Leipzig, Germany; 3 MoKi Analytics GmbH, Berlin, Germany; 4 Section of Pathology, Institute of Forensic Medicine, Jena University Hospital, Jena, Germany; 5 Klinik für Herz-und Thoraxchirurgie, Jena, Germany; 6 Microbiome Dynamics, Leibniz Institute for Natural Product Research and Infection Biology–Hans Knöll Institute (HKI), Jena, Germany; 7 State Key Laboratory of Microbial Resources, Institute of Microbiology, Chinese Academy of Sciences, Beijing, China; 8 College of Life Sciences, University of Chinese Academy of Sciences, Beijing, China; 9 Department of Molecular and Applied Microbiology, Leibniz Institute for Natural Product Research and Infection Biology - Hans Knöll Institute (HKI), Jena, Germany; 10 Moter Diagnostics, Berlin, Germany; 11 Friedrich Schiller University, Faculty of Biological Sciences, Jena, Germany; 12 Department of Medicine, The University of Hong Kong, Hong Kong SAR, China; 13 Cluster of Excellence Balance of the Microverse, Friedrich-Schiller-University Jena, Jena, Germany; 14 Leibniz Institute of Photonic Technology-Member of the research alliance “Leibniz Health Technologies”, Jena, Germany

**Keywords:** Microbial communities, Next-generation sequencing

## Abstract

Extensive investigations with sequencing methods demonstrate a large and diverse microbiome even in profound areas of the lung. However, there is still substantial lack of cultivation-based evidence and of the viability of the resident microorganisms. We collected human tissue specimens obtained from various regions during lung transplantations and from operations in distal alveolar areas. We characterized the samples by histology and applied sequencing, culture and imaging methods. Sequencing data detected the following trends in the composition of the microbiome: (i) From proximal to distal samples we observed a bacterial shift from *Staphylococci*, *Streptococci* and *Corynebacteria* towards preferentially anaerobically growing bacteria; (ii) we found large variations between individual patients regarding the detected bacterial genera. Culture and imaging methods revealed almost no viable microorganism in the distal alveolar regions in these patients. Bacterial signals detected by sequencing in the lung are likely due to very low numbers of bacteria and/or their remnants.

## Introduction

The human lung, for long thought to be a sterile environment, has recently been shown through advancements in scientific techniques, notably high-resolution sequencing methods, to harbor a hidden microbial landscape^1,2^. Previous studies reported a diverse and broad lung microbiome, capable of influencing numerous processes within lung tissue, including the development of lung diseases and infections. Current insights suggest that the upper airways predominantly serve as the primary source of microorganisms contributing to the microbiome of the lower airways^3^. The oral microbiome resembles in many parameters the deep lung microbiome, primarily characterized by the prevalence of *Bacteroidetes* and *Firmicutes*^4^. Microbial translocation apparently occurs through micro-aspiration, a regular phenomenon observed during sleep in individuals with sound health^5^. However, despite this process, the microbial biomass in the deep regions of the healthy lung remains low compared to other anatomical sites, particularly the nasopharyngeal area^4^.

Numerous theories and hypotheses exist regarding the influence of the lung microbiome on respiratory health. Previous publications indicate that the lung microbiome plays a significant role in preserving the overall health of the respiratory tract^2^. It has been demonstrated that specific microbial communities can enhance lung health and resilience against infections and even protect the lung against infection. Some commensal bacteria, such as *Lactobacilli*, have been tested and provided a barrier against pneumococcal colonization in a respiratory dysbiosis model^6^.

Moreover, chronic respiratory conditions like bronchial asthma and chronic obstructive pulmonary disease (COPD) have been linked to distinct modifications of the lung microbiome. Specifically, a decline in bacterial diversity and the prevalence of *Gammaproteobacteria* have been correlated with disease exacerbation^3,7^.

Conversely, the microbiome of the respiratory tract may serve as a reservoir for potential infections. Certain facultative pathogenic microorganisms, characterized by high virulence, can indeed form part of the respiratory tract microbiome. For example, *Streptococcus pneumoniae* (*S. pneumoniae*) and *Staphylococcus aureus* (*S. aureus*) are prominent examples of bacteria known to colonize the nasopharyngeal area in numerous healthy individuals^8,9^. In case of a translocation from the epithelial surface into the tissue these bacteria can turn from harmless colonizers to pathogens and induce severe invasive infections, including pneumonia^10^. Yet, invasive tissue infections require multiplying and metabolically active bacteria that grow and express exoproteins and toxins that destroy tissue and overcome the immune system^11,12^. Consequently, it is very relevant whether bacteria are in a metabolic active stage or only present as dead remnants in the respiratory microbiome.

The majority of the studies concerning the lung microbiome are based on culture-independent methods^3,13^. These methods include next-generation sequencing (NGS) and whole metagenome shotgun sequencing (WMGS) that likewise detect live and dead microorganisms^14^. The studies suggest the lung as a habitat for a diverse and intricate assembly of microorganisms. The applied methods, however, have some disadvantages, as (i) the low biomass of the lung microbiome results in considerable background noise in sequencing data and (ii) the data do not provide information on the viability and the virulent capacity of the microorganisms^14,15^. Interestingly, new methods to detect viable bacteria within the lungs are currently described, such as magnetic-activated cell sorting (MACS) that show distinct bacterial genera^16^. Yet, only a few studies are available that analyze the microbiome of the lung by culture methods, where selectively live and growing microorganisms are discovered^15,17^. Some of the culture-based studies were performed with murine lungs and typical murine strains were detected^17^. The lung microbiome in mice differs from that in humans, as distinct bacteria colonize the nasopharyngeal areas and constitute the lung microbiome^17,18^. The situation in mice is different to the human situation, since the anatomical structures are much smaller and contaminations can occur easier^19^.

Human studies that analyze the lung microbiome in small and deep airways are rare and mainly done by bronchoalveolar lavage (BAL), as the sampling of the lower respiratory tract is technically difficult^20^. Sequencing data reveal that bacterial DNA density becomes extremely low in smaller airways. Data obtained by culturing bacteria are difficult to obtain due to the very low bacterial numbers and the risk of contamination via the bronchoscopy sampling process^21,22^.

To further validate the large amount of -omics data generated in recent years, we need to improve culture methods to compare these data and understand bacterial virulence and adaptation mechanisms to the human lung environment. Despite the technical limitations and associated ethical issues in humans, we could overcome these limitations by analyzing human specimens obtained from different lung regions during surgery and lung transplantation by sequencing, culture, and imaging methods. In parallel to sequencing analysis, we used extended culture methods with different types of aerobic and anaerobic agar plates, blood culture flasks and prolonged incubation times for up to 21 days. Imaging methods include histological analysis as well as fluorescence in situ hybridization (FISH). In this study, we aimed to compare sequencing data with culture and imaging results to better understand the impact of the large amount of bacteria detected by sequencing method.

## Results

### Bacterial biomass and viability decrease from proximal to distal sites in lung transplantation samples

The upper respiratory region, encompassing the nasal and tracheal areas, is known for its abundant microbial flora^23^. In some reviews the vocal cords in the larynx have been defined as the line between the upper and lower respiratory tract^21^. To precisely analyze the decline of microorganisms according to the anatomical location within the lung, we collected samples from 3 whole lungs that were explanted during lung transplantation (patient 1–3). We collected samples from the main bronchus, the lobar bronchus, the segmental bronchus and from the distal bronchus near alveolar areas in order to compare proximal to distal areas of the lung. The patients undergoing lung transplantation suffered from different diseases that had compromised their lung function with a possible effect on the lung microbiome. Nonetheless, we recovered bacterial colonies by culture methods from the main, lobar, and segmental bronchus, but not from distal, alveolar samples (Table. 1, Supplementary Table 1, Supplementary Fig. 1). To verify the localization of the samples obtained from lung transplantation we further performed a histological analysis (Table. 2). As demonstrated, we observed mainly bronchial structures in the proximal bronchi, whereas in the distal regions high rates of alveolar structures were present.

**Table 1:** Overview of the results of the various analysis methods of tissue samples from 3 patients obtaining lung transplantations

Patient No.	Samples	Culture	FISH (activity of bacteria)	DAPI (amount of bacteria)
1	Main bronchus	-	-	-
	Lobar bronchus	-	-	-
	Segmental bronchus	-	-	-
	Distal bronchus	-	-	-
2	Main bronchus	-	-	few
	Lobar bronchus	*- S. aureus* *- C. aurimucosum* *- S. oralis*	positive	few
	Segmental bronchus	-	-	few
	Distal bronchus	-	-	few
3	Main bronchus	- *S. aureus*	-	-
	Lobar bronchus	-	-	few
	Segmental bronchus	- *S. aureus*	-	few
	Distal bronchus	-	-	-

Culture was performed to detect live bacteria. FISH was used for the detection of bacterial activity. DAPI was used for the detection of the number of bacteria.

**Table 2:** Histopathological determination of lung samples from 3 lung transplants and classification into alveolar or bronchial lung tissue

		Histopathological classification in %	
Patient No.	Sample side	Bronchial region	Alveolar region
1	Main bronchus	99	1
	Lobar bronchus	99	1
	Segmental bronchus	45	55
	Distal bronchus	15	85
2	Main bronchus	100	0
	Lobar bronchus	45	55
	Segmental bronchus	70	30
	Distal bronchus	0	100
3	Main bronchus	100	0
	Lobar bronchus	2	98
	Segmental bronchus	1	99
	Distal bronchus	3	97

### Multiple phyla are detected by sequencing in all lung areas with a shift to anaerobic bacteria in the distal bronchial/alveolar regions

**Fig. 1:**
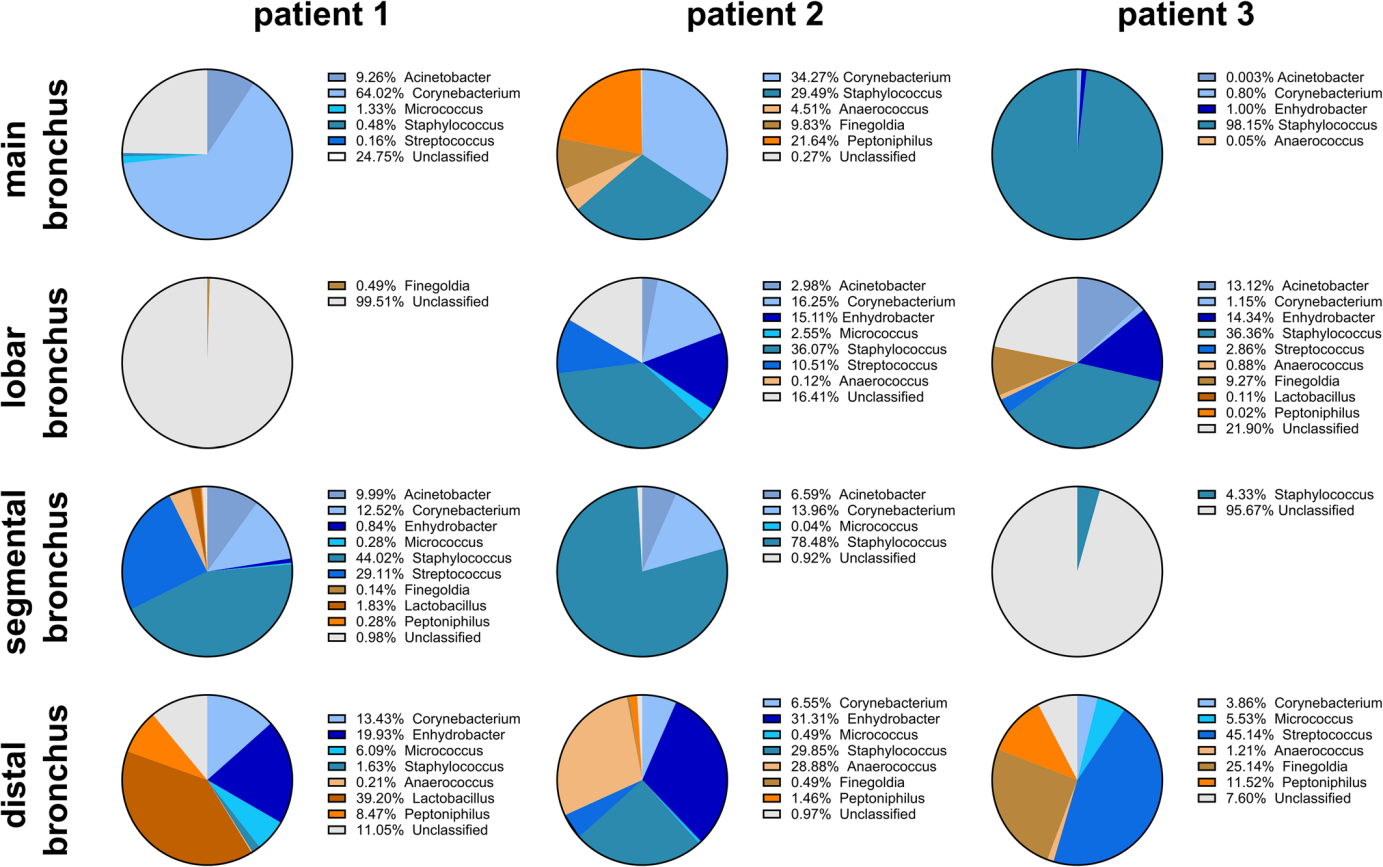
Detection of bacterial genera in four different lung regions. Sequencing of bacterial DNA was performed from the main, the lobar, the segmental and the distal bronchus from 3 patients (transplantation lungs). In the main, lobar and segmental bronchi we detected *Staphylococcus* and *Corynebacterium* with the highest prevalence. In the distal bronchi we found a high rate of mainly anaerobically growing genera, such as *Lactobacillus*, *Anaerococcus* or *Finegoldia*.

In contrast to the results from culture and imaging analysis, next-generation DNA sequencing (NGS) revealed multiple phyla in all areas analyzed (Fig. 1). In the main bronchus, *Staphylococcus* and *Corynebacterium* were detected with the highest prevalence. The same genera could be found in the lobar and segmental bronchi. In the distal bronchial/alveolar regions we found a different bacterial distribution. We still detected *Staphylococci* and *Corynebacteria*, but we also found a high number of mainly anaerobically growing bacteria, such as *Lactobacillus*, *Anaerococcus* or *Finegoldia* that made up to 50% of the discovered microorganisms. This effect was eminent in all transplanted patients.

**Fig. 2:**
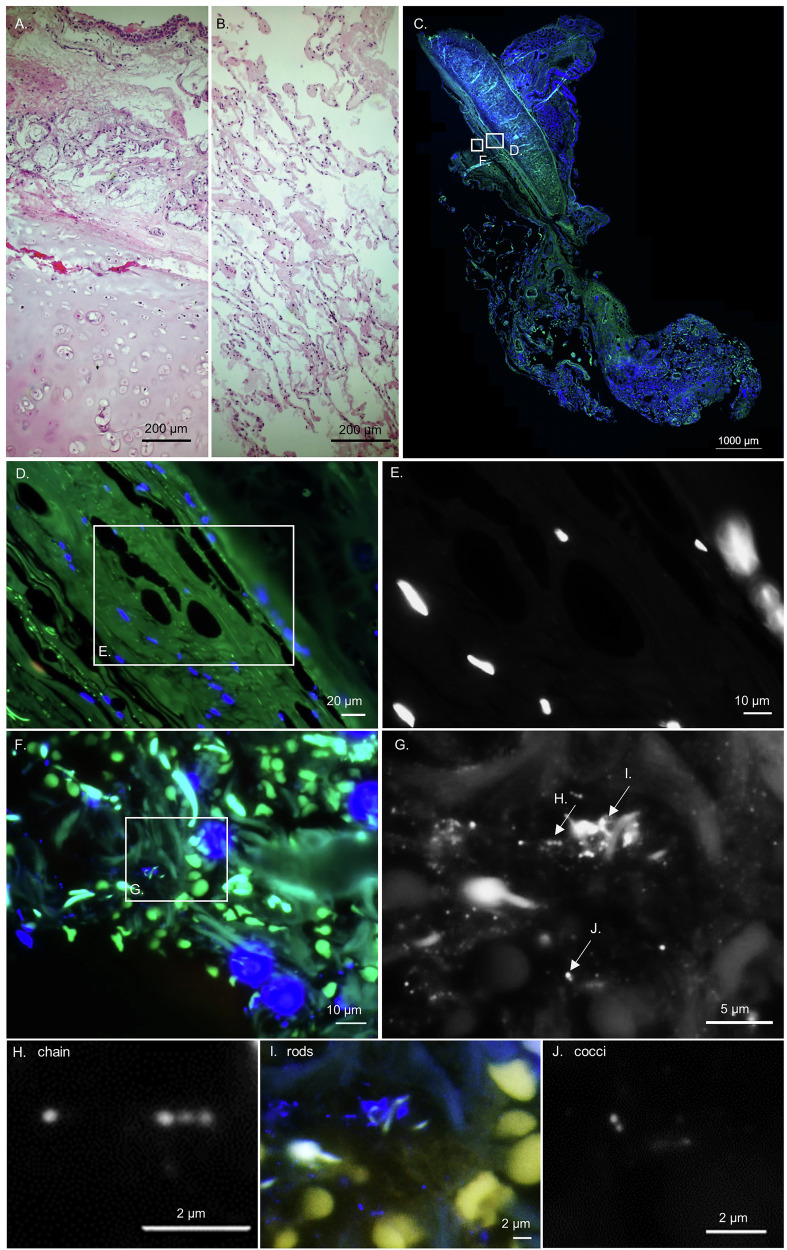
Visualization of the lung samples by histology and FISH-analysis. **A**, **B** Histological findings of the bronchus and alveolar parenchyma with unremarkable and local cellularity without any increase in inflammatory cells. **A** Bronchial parenchyma (transplantation patient 1), **B** alveolar parenchyma (transplantation patient 1). HE staining; scale: 200 µm. **C**–**J** FISH analysis of lung tissue, showing no or few bacteria. FISH of transplantation patient 2, hybridized with the pan-bacterial FISH probe EUB338 in orange and the nonsense control FISH probe NON338 in magenta combined with the nucleic acid specific stain DAPI in blue. Autofluorescence of the tissue background is shown Ldiin green. **C** Overview of the entire lung tissue sample (tissue background in green and DAPI in blue). **D** Higher magnification of the inset marked in (**C**) (tissue background in green and DAPI in blue), exemplarily showing lung tissue without microorganisms. **E** Higher magnification of the inset marked in (**D**): only the DAPI channel is shown (black and white) featuring host cell nuclei, but no bacteria. **F** Higher magnification of the inset marked in (**C**) (tissue background in green and DAPI in blue), exemplarily marking one of the few regions in the tissue, where single bacteria were found. **G** Higher magnification of inset marked in (**F**), which exemplarily shows a tissue region with single bacteria: only the DAPI channel is shown (black and white), featuring single bacteria of different morphotypes (arrows). **H** DAPI (black and white) stain showing cocci in a chain. **I** Overlay of the pan-bacterial FISH probe EUB338 in orange, the nonsense control FISH probe NON338 in magenta, the nucleic acid stain DAPI in blue and the tissue background in green showing rod shaped bacteria. **J** DAPI (black and white) stain showing single cocci.

Beside NGS, we performed FISH-analysis to locate bacteria within the different lung samples (Fig. 2). Interestingly, bacteria with different morphotypes were detected.

Overall, in samples obtained from lung transplantation, viable and dividing bacteria were only detected in the bronchial regions, whereas the distal bronchi/alveoli largely lacked culturable and morphological intact looking bacteria. Microbial DNA was detectable by sequencing methods in all lung regions. In the proximal bronchial regions we found mainly bacterial DNA from skin-colonizing bacteria, such as *Corynebacteria* and *Staphylococci*. In the distal bronchial/alveolar regions we found a remarkably changed profile dominated by anaerobic bacteria.

### Next-generation sequencing reveals a high diversity of different phyla, including typical anaerobic bacteria, in distal alveolar regions

**Fig. 3:**
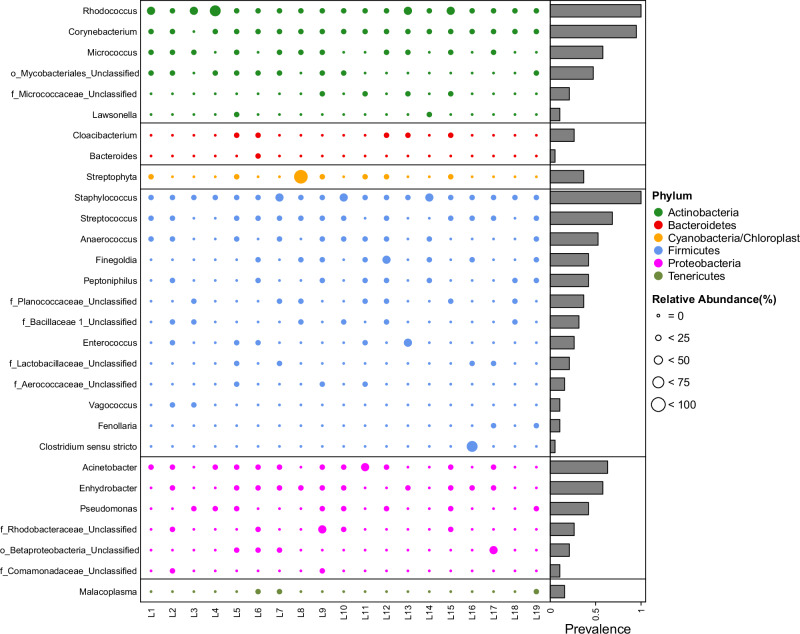
Detection of bacterial genera in lung tissues from 19 patients by next-generation sequencing. A Bubble charts presenting the relative abundance of the top 30 taxa in the lung samples of 19 patients (L1-L19). The taxa are classified down to genus-level or the lowest classifiable taxonomic level (f, o, and p represent family, order, and phylum, respectively). The size of bubble indicates the relative abundances (%) of each taxon. Bubbles are colored based on the corresponding phylum level. The additional bar plot in the right-most column shows the prevalence of taxa in healthy samples. * Genus has been observed in cultivation of healthy samples.

High throughput DNA sequencing (NGS) followed by composition and diversity analyses was used to decipher the microbiome in human lung samples. We detected multiple signals in all samples analyzed from 19 patient samples (L1-L19) The microbiome of all 19 lung samples was dominated by *Rhodococcus*, *Corynebacterium*, and *Staphylococcus* with a prevalence of 1 (Fig. 3). Additionally, we provide the relative abundance of the top genera in each lung sample (Supplementary Fig. 2). These results are in line with the data obtained from the bronchial and alveolar samples from patients after lung transplantation. The detailed analysis of peripheral alveolar samples also reveals the presence of anaerobic bacteria, such as *Fingoldia* that were in some samples dominant, such as in sample L12 (Fig. 3, Supplementary Fig. 2). Nevertheless, in the 19 alveolar samples obtained from different patients, we notice a high interindividual diversity, including *Rhodococcus*, *Corynebacterium*, *Staphylococcus* and *Acinetobacter*. While *Acinetobacter* and *Enhydrobacter* are frequently found in environmental settings, they are also known to colonize the human respiratory tract^24–26^.

### Distal lung samples were largely devoid of culturable and living bacteria

**Fig. 4:**
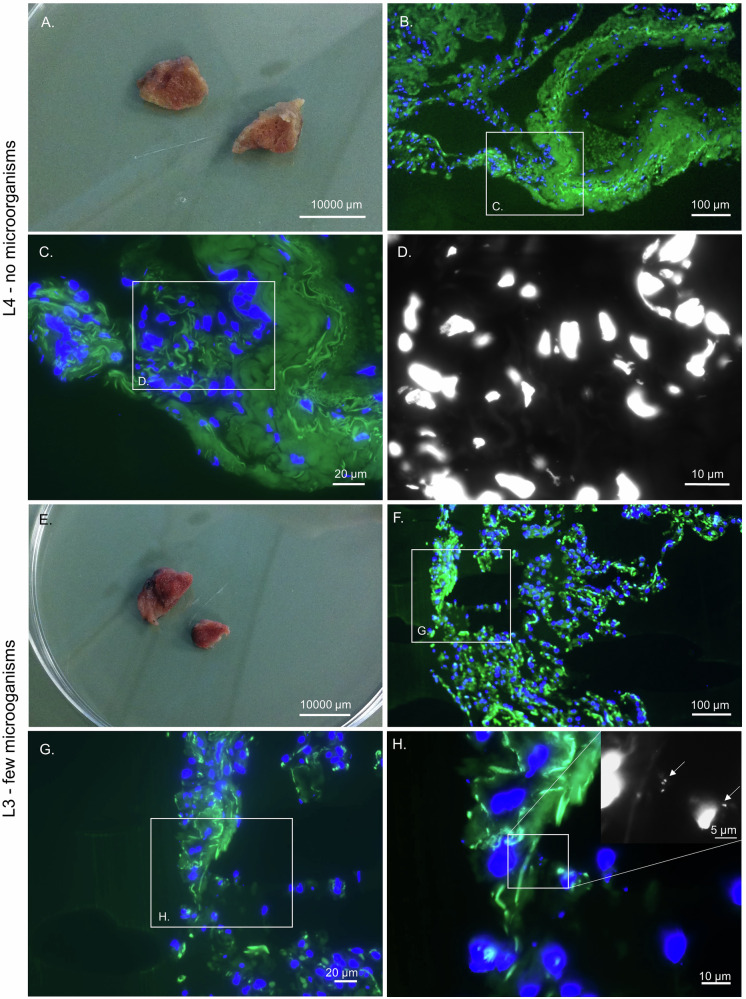
Visualization of peripheral lung samples by FISH-analysis. Fluorescence in situ hybridization (FISH) of lung tissue, exemplary images for negative and positive findings of bacteria. FISH of histological sections of L4 and L3. In FISH, the autofluorescent tissue background is shown in green, nucleic acids are stained by DAPI and shown in blue. The sections were screened for FISH-positive bacteria with the pan-bacterial FISH probe EUB338 in orange and the nonsense control FISH probe NON338 in magenta (not shown). **A** Macroscopic impression of the lung sample L4 before embedding and sectioning. **B** Microscopic overview of the lung tissue. **C** Higher magnification of the inset marked in (**B**) featuring host cell nuclei in blue and tissue background in green. **D** Higher magnification of the inset marked in (**C**) showing the DAPI channel in black and white: no microorganisms were found in this lung tissue sample. **E** Macroscopic overview of the lung sample L3 before embedding and sectioning. **F** Microscopic overview of the lung tissue. **G** Higher magnification of the inset marked **F** with the nucleic acid stain DAPI in blue and the tissue background in green. **H** Higher magnification of the inset marked in (**G**): few single DAPI positive microorganisms were visualized (shown in black and white in the inset marked with an arrow). No signal was detectable with the FISH probe EUB338.

In order to gain a deeper understanding of the microbiome in the distal lung, we investigated 19 lung samples from the alveolar regions, collected from patients undergoing lung surgery (Table. 3). By histological analysis we confirmed that the samples were mainly composed of alveolar structures (70–100%; Table. 4) and represent distal alveolar regions. All patients suffered from different pulmonary diseases requiring surgical intervention and all patients received antibiotics (Supplementary Table 2). The samples were taken from morphological healthy tissue, located as far as possible from the pathological processes that was confirmed by histopathological examination, as described in Material and Methods. After sterile extraction of the lung samples, a defined set of culture methods, consisting of different types of aerobic and anaerobic culturing methods and preincubation in blood culture flasks, was used (Supplementary Fig. 1). Additionally, all samples were analyzed by FISH-technology to visualize viable and dividing bacteria (Fig. 4).

**Table 3:** Overview of the results of the various analysis methods of the individual samples from 19 human lung tissues

Sample	Culture	FISH (activity of bacteria)	DAPI (amount of bacteria)
L1	*S. aureus*	-	-
L2	**-**	-	-
L3	**-**	-	few
L4	**-**	-	-
L5	**-**	-	-
L6	**-**	-	-
L7	**-**	-	-
L8	**-**	-	-
L9	**-**	-	-
L10	**-**	-	few
L11	**-**	**-**	-
L12	**-**	**-**	-
L13	*K. pneumoniae, K. variicola*, *K. rhizophila*	**-**	-
L14	**-**	**+**	few
L15	**-**	**-**	-
L16	**-**	**-**	-
L17	**-**	**-**	few
L18	**-**	**-**	-
L19	**-**	**-**	-

Culture was performed to detect live bacteria; FISH was used for the detection of bacterial activity and DAPI for the detection of the number of bacteria.

**Table 4:** Histopathological determination of lung samples from distal lung tissue and classification into alveolar or bronchial lung tissue

	Histopathological classification in %	
Patient No.	Alveolar region	Bronchial region
**L1**	99	1
**L2**	70	20
**L3**	85	15
**L4**	100	0
**L5**	99	1
**L6**	99	1
**L7**	98	2
**L8**	95	5
**L9**	90	10
**L10**	100	0
**L11**	90	10
**L12**	98	2
**L13**	97	3
**L14**	99	1
**L15**	99	1
**L16**	100	0
**L17**	93	7
**L18**	82	18
**L19**	99	1

By using Hematoxylin-Eosin staining, lung tissue could be classified into specific lung areas.

Interestingly, solely in two out of 19 samples we detected growing bacteria by bacterial culture methods (Table. 3). In sample L1, *S. aureus* was detected by culture. *S. aureus* is recognized as a common colonizing bacterium of the skin and nasopharyngeal area. In addition, *Klebsiella* and an environmental bacterium *Kocuria rhizophila* were detectable in sample L13. *Klebsiella* are known as the causative pathogens of hospital-acquired pneumonia^27^. Indeed, the patient suffered from a larynx-carcinoma treated by a laryngectomy that potentially resulted in a missing barrier to protect the lung from colonizing and environmental microorganisms (Supplementary Table 2). Imaging methods suggest in some samples the presence of very few DAPI positive microorganisms, whereas growing and dividing bacterial figures were not visible (Table. 3, Fig. 4).

## Discussion

The investigation of the lung microbiome is associated with challenges when it comes to identify and understand the proposed diverse microbial populations that are reported to inhabit the airways. Historically, the deep lung regions were considered sterile; however, advancements in sequencing technologies have unraveled a complex array of bacteria, viruses, fungi, and archaea inhabiting this niche^23^. While the lung microbiome is less diverse compared to other body sites, it nevertheless could influence the local immune responses, respiratory physiology, and disease susceptibility^2^.

The reliance on sequencing techniques rather than cultivation methods poses a significant challenge in characterizing the viability and activity of identified bacteria within the lung microbiome^28^. This disparity raises uncertainties regarding whether the detected genetic material represents living, metabolically active bacteria or simply remnants of non-viable organisms. Sequencing approaches, while powerful in elucidating microbial diversity and community structure, primarily capture genetic signatures and do not inherently distinguish between live, dormant, or dead microorganisms. Consequently, the inability to differentiate between viable and non-viable bacteria complicates the interpretation of their functional roles and actual contributions to the lung microenvironment. This discrepancy between sequencing-based identification and cultivation-based assessment leads to an ambiguity in understanding the true physiological relevance of the identified bacterial taxa. It remains unclear whether the detected bacteria actively participate in lung homeostasis, immune modulation, or pathogenic processes, or if they represent transient or inactive members of the microbiome.

In our study we investigated human lung tissue, as a recent study demonstrated that whole lung tissue is the preferred sampling strategy in order to investigate the lung microbiome; hence, we did not analyze bronchoalveolar lavage^19^. We obtained lung tissue samples from patients requiring lung transplantation and samples collected from the lungs of patients who underwent surgical intervention due to lung pathology. The analyzed materials were either obtained from a surgical procedure or a lung transplantation. Prior to the operation, the patient received preoperative antibiotic prophylaxis (Supplementary Table 1, 2). Administration of beta-lactam antibiotics potentially leads to a reduction of some bacterial genera. However, in most patients the beta-lactam antibiotic cefazolin was used that is characterized by a very narrow spectrum on Gram-positive bacteria. Actually, the staphylococci and streptococci found in lung transplantation patients were tested sensitive to cefazolin, notwithstanding that the patients received antibiotics. Therefore, this finding suggests that systemic treatment with antibiotics did not eradicate the microbial flora in the lung.

All samples were examined by using state-of-the-art bacteriological methods to identify the growth of aerobic and anaerobic microorganisms. Here, we isolated cultivable bacteria mainly from the bronchial regions, and identified typical bacteria (e. g. *Staphylococcus*, *Streptococcus* and *Corynebacteria*). Remarkably, we largely failed to isolate living and growing bacteria from the distal alveolar areas. These results were confirmed by FISH-analysis that did not reveal actively dividing bacteria in the distal regions of the lung. Only from two out of 19 samples, cultivation of bacteria was possible from alveolar samples. Interestingly, both patients from which these samples derived were diagnosed with a severe disease that led to translocation of colonizing bacteria to the lower airways (Supplementary Table 2). Taken together, these results suggest that the lower respiratory airways and particularly the alveolar regions are largely free of metabolically active and growing bacteria.

By contrast, DNA sequencing results indicated the presence of bacterial DNA in all lung areas. The DNA sequences mainly covered typical skin-colonizing bacteria, such as *Rhodococcus*, *Corynebacteria*, *Staphylococcus* and *Streptococcus*. (Fig. 1, Fig. 3). It was also interesting to notice that within the samples collected from transplantation patients, we observed a shift of the bacterial flora toward anaerobic bacteria in the distal regions that could be partly confirmed in the alveolar samples. These findings are in line with published literature that describe anaerobic bacteria as part of the lung microbiota, even in healthy individuals^29^. Yet, anaerobic bacteria are further described in different chronic respiratory diseases^30^. Interestingly, no strict anaerobic bacteria were detected here by using anaerobic culture methods, suggesting that anaerobic bacteria are at a stage difficult or even unable to be cultivated. These results are in line with previous results that demonstrate the presence of bacterial signs in the human lung mostly by culture independent methods^31,32^. The signals are most likely induced by bacterial remnants that arise from the upper respiratory tract and have been inhaled and reach the lower respiratory tract at a minor concentration^23^. During sleep, respiratory rates tend to decrease, potentially leading to a higher concentration of inhaled bacteria due to reduced clearance mechanisms and altered airflow patterns. Additionally, changes in body posture during sleep might influence the deposition of inhaled particles in the respiratory tract and contribute to the lung microbiome. Conversely, upon awakening, individuals often take deeper breaths and experience increased respiratory rates, which could facilitate the expulsion of retained microorganisms. This proposed mechanism suggests that the circadian rhythm, coupled with altered breathing patterns, might contribute to the inhalation and subsequent expulsion of bacteria from the respiratory tract. Notably, methods based on sequencing methods do not differentiate between live and dead bacteria, which is important to evaluate the virulent potential of the microbes^32^.

Our study has limitations that are largely associated with the difficulty to sample human lung specimens. (i) Firstly, the antibiotic given to the patients might have affected the microbiome of the lung. Although most patients only received the very narrow spectrum cefazolin, we cannot completely exclude a systemic effect on the lung microbiome. (ii) Furthermore, it is important to emphasize that the composition of the lung microbiome changes dynamically under the influence of various diseases^1^. This phenomenon was extensively described for cystic fibrosis patients^33^, but also other diseases, such as cancer or chronic lung diseases of our patients might influence the lung microbiome. (iii) The methods applied for routine diagnostics that we used to detect live and growing bacteria often failed to grow dormant bacterial phenotypes or other difficult to detect microorganisms. This is a clear limitation of our manuscript and a well-known problem in routine diagnostics. (iv) Due to ethical restrictions the sample size of our study is quite low. Nevertheless, our collections of patients´ specimens well reflect the comorbid hospitalized population, as we included patients with cancer and chronic inflammatory diseases. (v) Finally, due to the low numbers of colonizing bacteria we cannot exclude that environmental contaminants could interfere with our analysis.

Taking together, we performed an extensive analysis of the human lung microbiome by culture, imaging and sequencing methods. Our most striking result shows that the alveolar regions appear to be largely free of intact and actively growing bacteria and only harbor a very small number of bacteria. By contrast, sequencing methods detect a variety of bacterial genera most of them might represent bacterial remnants from the upper respiratory airways or bacteria that remain in a growth-arrested stage.

## Methods

### Sample collection and preparation

Lung tissue was obtained from 22 patients (3 lungs from lung transplantation and 19 lung samples (L1-L19) from elective intervention; ethical approval: 2020-1894-Material, 2020-1894_1-Material). Here, 3 lung transplants with 4 sample sites (Table. 1) and 19 elective lung interventions (Table. 3) were analyzed. All patients received perioperative antibiotics (Supplementary Table 1 and Supplementary Table 2). For the elective lung procedures, 2 g cefazolin and for the lung transplants 4.0 g piperacillin /0.5 g tazobactam were administered. The lung tissue from the elective interventions, a peritumoral parenchyma section was removed for further experiments. To ensure the R0 recession, the postoperative report and the assessment of the pathology were always consulted, so that only tumor-free lung tissue was used for further research purposes, which was the case for all used lung specimen.

The material from the lung transplantation includes 4 different sampling sites located along the bronchial line. Starting at the main bronchus moving in the direction of the periphery of the lungs, on a connecting line along the bronchial branches between these points 2 locations were selected, 1/3 away from the main bronchus and 1/3 away from the outside of the lungs (Fig. 1). All materials were collected under sterile conditions in the operating room.

The study was approved by the local ethics committee (UKJ: 2019-1519-2, 2020-1894-Material, 2020-1894_1-Material), Chair: Prof Ekkehard Schleußner, Jena University Hospital (ethikkommision@med.uni-jena.de).

### Bacterial culture identification

The cultivation of the lung specimen was started directly after extraction under sterile conditions at room temperature (Supplementary Fig. 1). The material was removed with tweezers and cut into smaller pieces with scissors to be transferred into a disposable mixing container ProbeAX (Axon Lab, Baden, Switzerland), which was then placed on the IKA Ultra-TurrAX® Tube Drive P control disperser (IKA-Werke, Staufen, Germany) for homogenization. Processing was carried out at 6000 rpm for 3 min. For all samples, this mixture, consisting of the NaCl solution and the comminuted tissue, was transferred to a 15 ml tube without the stainless-steel balls.

For the bacterial cultivation, different culture media was used. Columbia blood agar with sheep blood (Thermo Fisher Scientific, Dreieich, Germany), chocolate agar with Vitox (Thermo Fisher Scientific, Dreieich, Germany), double plate with Schaedler anaerobic agar/Schaedler anaerobic KV selective agar (Thermo Fisher Scientific, Dreieich, Germany) were each inoculated with 250 µl of the processed material. For incubation under anaerobic conditions, the divided Schaedler anaerobic agar/Schaedler anaerobic KV selective agar were placed in an Oxoid™ AnaeroJar™ 2.5 l container (Thermo Fisher Scientific, Dreieich, Germany), in which an additional Oxoid™ AnaeroGen™ 2.5 l sachet, one indicator strip Oxoid™ Resazurin Anaerobic Indicator (Thermo Fisher Scientific, Dreieich, Germany) and one of the 2 Brain Heart Infuses were added. The success of the reaction process under anaerobic conditions was checked once after 24–48 h, based on the color change of the indicator strip. Agar plates were incubated for 7 days at 36 °C ± 2 °C, 5% CO_2_ and 95% relative humidity (RH) in a Steri-Cult™ CO_2_ Incubator (Thermo Fisher Scientific, Dreieich, Germany). Colonies on a plate that could not be distinguished from one another had to be spread out again on fresh plates to obtain selective single colonies for further analyzing.

In addition, blood culture flasks were inoculated with the obtained material: BD BACTEC™ Plus Aerobic/F Culture Vials (BD, Heidelberg, Germany) and BD BACTEC™ Lytic/10 Anaerobic/F Culture Vials (BD, Heidelberg, Germany). The bottle heads were always disinfected with Sterilium (Hartmann, Heidenheim, Germany) before and after each use. 1 ml of the tissue mix was injected using a disposable cannula. Since the volume of the sample to be added was too small (< 3.0 ml) according to manufacturer’s protocol, 2 ml of BD BACTEC FOS™ were added as a supplement per flask. Both bottles were incubated in the BD BACTEC FX blood culture incubator (BD, Heidelberg, Germany) for a period of 21 days. Negative remaining bottles were being disposed; positively registered culture bottles were removed from the incubator after notification. A small amount of liquid from these bottles (~50 µl) was prepared for subculture under the same conditions as described, according to the agar plate protocol above.

To identify further bacteria, 250 µl of the material mix was inoculated into 2 BD BBL™ BHI Brain Heart Infusion 8 ml (BD, Heidelberg, Germany). One of the brain-heart infusions was placed in the MIR-162 Gravity Convection incubator (Sanyo, Moriguchi, Japan) at 36 °C ± 2 °C under aerobic conditions. The tube that was intended to be stored anaerobically was placed in the anaerobic pot as described above. Both infusions were incubated with the cap loosened for 7 days. If a bacterial growth was evident at the end of the observation period, 100 μl of the contents was pipetted on each of the usual 3 agar plates and further processed according to the agar plate protocol under the same conditions as described.

About 1750 μl of the remaining material from the NaCl solution and lung tissue mix was pipetted into a 2 ml micro tube, which was then stored in the −86 °C Forma™ 900 Series Ultra Low Freezer (Thermo Fisher Scientific, Schwerte, Germany) at around −80 °C ± 2 °C for further experiments.

All bacterial cultures were evaluated using the MALDI-TOF method in the VITEK® MS mass spectrometer (bioMérieux, Nürtingen, Germany). For this purpose, some colony material was picked up and transferred to a sample position of a target VITEK® MS-DS SLIDE (bioMérieux, Nürtingen, Germany). A 1 μl drop of the ready-to-use, light-stable matrix solution CHCA (bioMérieux, Nürtingen, Germany) was then pipetted onto this sample. After the mixture had dried, the targets could be inserted into the VITEK MS for measurement. The results are based on the comparison of the measured data with the mass spectra stored in the database.

### Fluorescence in situ hybridization (FISH)

In preparation for performing FISH-analysis, lung tissue samples were fixed using FISHopt® (MoKi Analytics, Berlin, Germany) and embedded in cold polymerizing resin according to the manufacturer’s instructions. Sections of the embedded lung samples were analyzed by FISH as described previously^34^. Briefly, sections were first screened with the pan-bacterial, 16S rRNA directed probe EUB338 and the nonsense probe (NON338) to exclude unspecific probe binding (probes from Biomers, Ulm, Germany)^35,36^. The nucleic acid stain DAPI (4’,6-diamidino-2-phenylindole, Merck, Darmstadt, Germany) was applied as a counterstain to visualize host cell nuclei and microorganisms that contain too few ribosomes to be visualized by microscopy. A fourth microscopic channel was left without fluorochrome in order to control autofluorescence of the lung tissue. Upon detection of microorganisms, a panel of FISH probes was applied for identification on a genus- or species-specific level^37^. FISH image acquisition was performed with an epifluorescence microscope (AxioImagerZ2; Carl Zeiss, Jena, Germany, and Metafer; MetaSystems, Altlussheim, Germany) equipped with narrow band filter sets (AHF-Analysentechnik, Tübingen, Germany).

Each hybridization experiment was controlled using positive reference strains and negative control strains with minimum mismatches in the probe target sequence.

### Histological staining of lung samples

After FISH analysis, lung tissues were used for histological staining. For this, plastic was removed, the 2 µm thick sections were incubated in 2-methoxyethyl acetate, and transferred to an aqueous medium. Subsequently, routine staining of the sections using Hematoxylin and Eosin (HE) was performed.

### Sequencing

The DNA preparation from tissue was done commercially (DNA Sense, Aalborg, Denmark) by using two kits, Molzym Ultra­Deep Microbiome kit (Molzym, Bremen, Germany) and DNeasy Power Soil Pro kit (Qiagen, Hilden, Germany).

Briefly, for the Molzym Ultra­Deep Microbiome kit, the homogenized human lung sample (0.3–0.5 cm^3^) was used for DNA extraction using the Ultra­Deep Microbiome kit according to the manufacturer’s recommendations with minor modifications: initial proteinase K digestion was extended from 10 to 20 min, centrifugation steps were at 15,000 × *g*, and final elution was done in 40 µl of deionized water heated to 70 °C. After the host DNA depletion step, the pellet was re-suspended in 1 ml RS buffer and was split in two with one half used for fungi DNA extraction (DNeasy Power Soil Pro kit). The remaining half was continued, and these DNA extractions were used for bacteria/archaea 16S rRNA gene hypervariable region 4 (abV4­C) PCR amplification and sequencing. Gel electrophoresis using Tapestation 2200 (Genomic DNA and D1000 screentapes, Agilent, Santa Clara, USA) was used to validate product size and purity of a subset of DNA extracts. DNA concentration was measured using Qubit dsDNA HS Assay kit (Thermo Fisher Scientific, Waltham, USA).

For the DNeasy Power Soil Pro kit, half of the host DNA depleted samples in 0.5 ml Molzym Ultra­Deep Microbiome RS buffer were pelleted at 15,000 × *g* and used for DNA extraction with the DNeasy Power Soil Pro kit following the manufacturer’s recommendations with minor modifications. Gel electrophoresis using Tapestation 2200 (Genomic DNA and D1000 screentapes, Agilent, Santa Clara, USA) was used to validate product size and purity of a subset of DNA extracts. DNA concentration was measured using both Nanodrop and Qubit dsDNA HS Assay kit (Thermo Fisher Scientific, Waltham, USA).

### Sequencing library preparation

Amplicon libraries for the bacteria/archaea 16S rRNA gene hypervariable region 4 (abV4C) were prepared by a custom protocol based on an Illumina protocol. Up to 10 ng of extracted DNA was used as template for PCR amplification of the taxonomic marker genes. Each PCR reaction (25 μl) contained (12.5 μl) PCRBIO Ultra mix (PCR Biosystems, London, United Kingdom) and 400 nM of each forward and reverse tailed primer mix. PCR was done with the following program: Initial denaturation at 95 °C for 2 min, 30 cycles of amplification (95 °C for 15 s, 55 °C for 15 s, 72 °C for 50 s) and a final elongation at 72 °C for 5 min. Duplicate PCR reactions were performed for each sample and the duplicates were pooled after PCR. The forward and reverse, tailed primers targeting the bacteria/archaea 16S rRNA gene hypervariable region 4 (abV4C): [515FB] GTGYCAGCMGCCGCGGTAA and [806RB] GGACTACNVGGGTWTCTAAT^38,39^. The primer tails enable attachment of Illumina Nextera adaptors (Nextera XT DNA Library Preparation Kit, Illumina, San Diego, USA) necessary for sequencing in a subsequent PCR. The resulting amplicon libraries were purified using the standard protocol for CleanNGS SPRI beads (CleanNA, Waddinxveen, NL) with a bead to sample ratio of 4:5. DNA was eluted in 25 μL of nuclease free water (Qiagen, Hilden, Germany). DNA concentration was measured using Qubit dsDNA HS Assay kit (Thermo Fisher Scientific, Waltham, USA). Gel electrophoresis using Tapestation 2200 and D1000/High sensitivity D1000 screentapes (Agilent, Santa Clara, USA) was used to validate product size and purity of a subset of sequencing libraries. Sequencing libraries were prepared from the purified amplicon libraries using a second PCR. Each PCR reaction (25 μl) contained PCRBIO HiFi buffer (1x), PCRBIO HiFi Polymerase (1 U/reaction) (PCR Biosystems, London, UK), adaptor mix (400 nM of each forward and reverse) and up to 10 ng of amplicon library template. PCR was done with the following program: Initial denaturation at 95 °C for 2 min, 8 cycles of amplification (95 °C for 20 s, 55 °C for 30 s, 72 °C for 60 s) and a final elongation at 72 °C for 5 min. The resulting sequencing libraries were purified using the standard protocol for CleanNGS SPRI beads with a bead to sample ratio of 4:5. DNA was eluted in 25 μl of nuclease free water.

### DNA sequence analysis

The 16S rRNA gene sequencing data were processed and annotated by Lotus2 (v.2.24) with default parameters (cluster method: UPARSE). Contaminant OTUs were identified and filtered from the dataset using the prevalence-based method in the R package decontam (v.1.18.0)^40^. with default parameters. This method works by comparing the prevalence of each OTU in the samples to its prevalence in the negative controls (derived from DNA extraction and PCR steps). Any OTU exhibiting a significantly higher prevalence in the negative controls (defined by a *p*-value of less than 0.1) was flagged as a contaminant and subsequently removed. Given the possibility of contamination, despite the absence of Cyanobacteria/chloroplast sequences in the blank control, we removed all associated OTUs. To further avoid the contamination from mitochondria, each OTU sequence identified as human mitochondria sequences by blast analysis was also discarded.

The relative abundances were calculated based on the count results of Lotus2. Only the top 30 abundant genera were used for R package ComplexHeatmap to draw the bubble chart^41^. The core genera in transplanting samples were filtered using prevalence thresholds of 50%, and their mean relative abundance was shown as pie charts (Fig. 1).

The 16S rRNA sequencing data are available within the European Nucleotide Archive (ENA, accession number PRJEB72001, Supplementary Table 3). Read count of operational taxonomic units (OTUs) across all samples.

## Supplementary information

Supplementary information

Supplementary Data

## Data Availability

The 16S rRNA sequencing data are available within the European Nucleotide Archive (ENA, accession number PRJEB72001).
