# Supplementary material for: The human lung microbiome progressively diminishes in the distal alveolar regions
Source: NPJ Biofilms Microbiomes. 2026 Jun 15;12:124. doi: 10.1038/s41522-026-01047-y (PMC13287667; doi:10.1038/s41522-026-01047-y)
Supplement: Supplementary file 1 — Supplementary information [file 41522_2026_1047_MOESM1_ESM.pdf]

Supplementary information

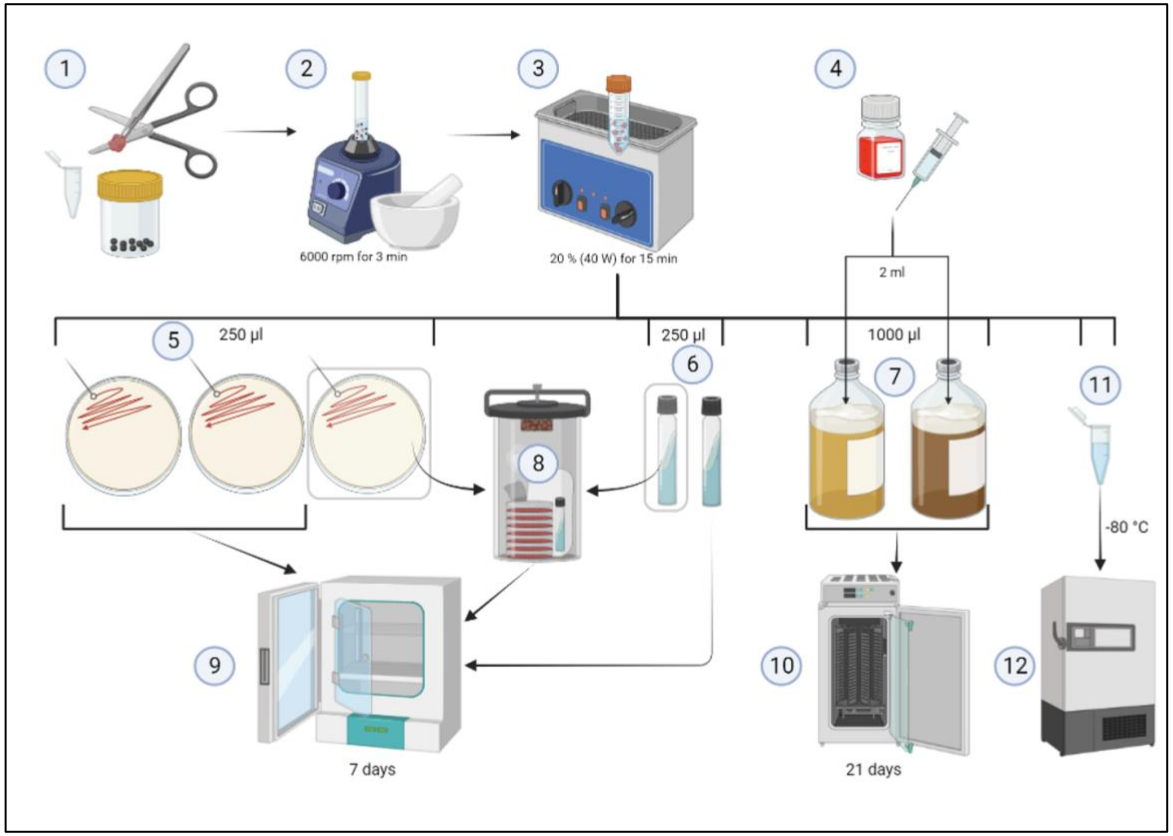

Supplementary Figure 1. Cultural processing of the lung specimen. (1) Cutting of the sample into smaller pieces to be transferred into a disposable mixing container ProbeAX **(2)** Homogenization with the IKA Ultra-TurrAX® Tube Drive P control disperser with a speed of approximately 6,000 rpm for 3 min. This work step was only temporarily replaced for the samples of 3 patients (LuMi19, LuMi20, LuMi21 samples a-d) by using a mortar and pestle **(3)** Sonification in BS 14 Bactosonic ultrasonic bath at the lowest power (20 % =40 W) for 15 min **(4)** Supplementation by adding 2 ml of

BD BACTEC FOS™ per flask **(5)** Inoculation of 3 Agar Plates each with 250 µl of the processed material **(6)** Inoculation of 2 Brain Heart Infusion each with 250 µl of the processed material **(7)** Inoculation of 2 different blood culture flasks (aerob/anaerob) each with 1000 µl of the processed material **(8)** Transfer of 1 Brain Heart Infusion and the Schaedler double agar plate into an Anaerobic jar **(9)** Incubation for 7 days in Incubator **(10)** Incubation for 21 days in BD BACTEC FX blood culture incubator **(11)** Pipetting of the remaining material into a 2 ml micro tube **(12)** Storage in the -86 °C Ultra Low Freezer for further experiments.

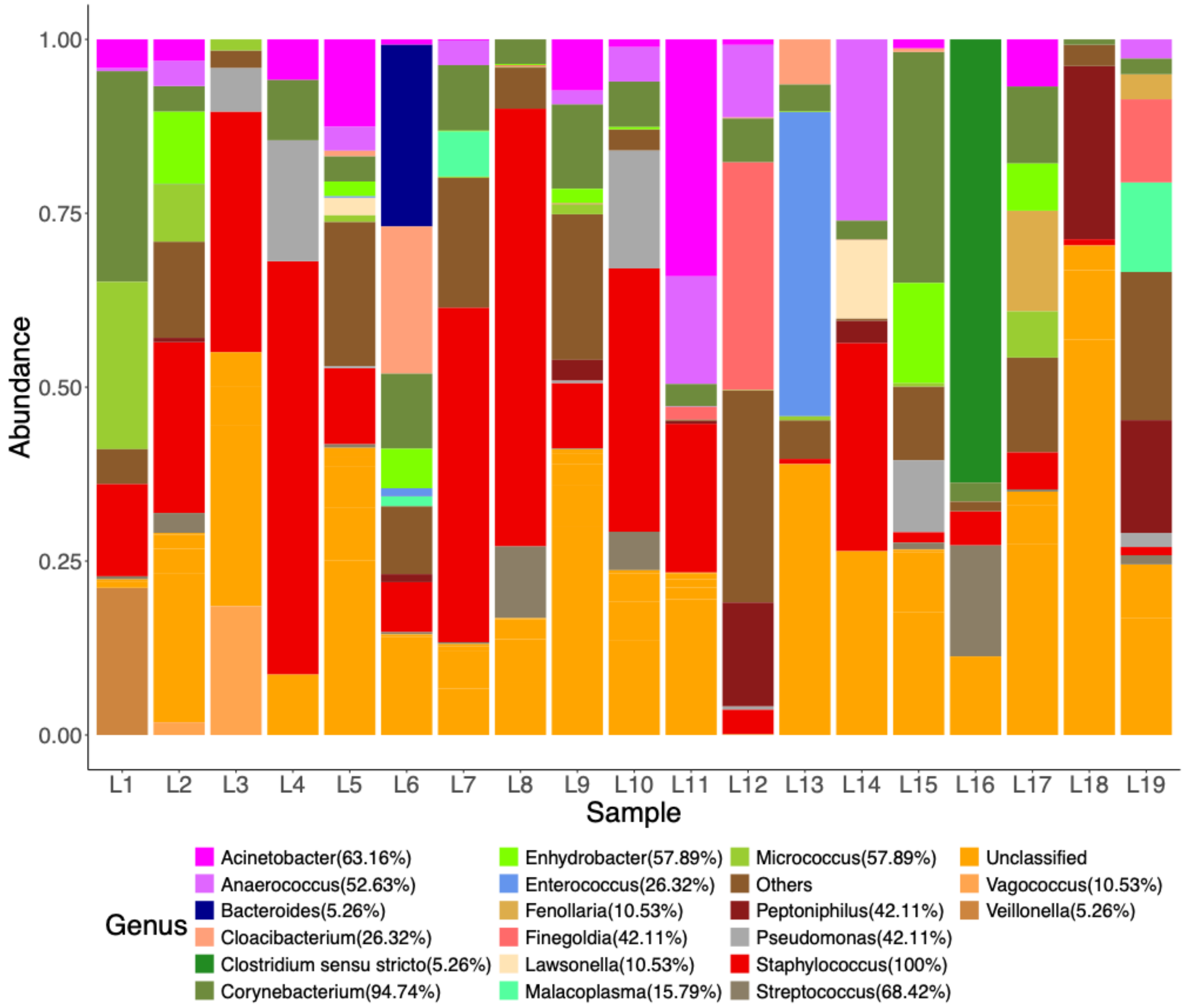

Supplementary Figure 2. Relative abundance of the top 20 abundant genera in each healthy lung sample. Lung samples from 19 patients (L1-L19) with percentage of samples in which the genus was detected (prevalence).

Supplementary Table 1. Overview of previous illnesses, medications or therapies of lung transplantation patients. Listed are the results of the bronchoalveolar lavage (BAL), which would take place immediately before lung transplantation. In addition, the antibiotics taken preoperatively at least up to 1 month before the day of the are summarized.

**Patient**	**Preexisting Diseases**	**BAL before lung transplantation**	**Antibiotics before surgery**
**1**	- Idiopathic pulmonary fibrosis with exacerbation, ID 11/2020<br>- high-dose oral steroid therapy: Prednisolon 20 mg/d, for about 2 years<br>- chronic partial respiratory failure and restrictive ventilation disorder, ID 11/2020<br>- Atypical wedge resection of left upper lobe (01/21)<br>- Long-term oxygen therapy (since 12/20)<br>- Bronchial asthma (ID 2005), free of symptoms for years<br>- Arterial hypertension (ID 2000)<br>- Leukoplakia vocal folds on both sides (no indication of malignancy, atypical squamous epithelial mucosa, superficial necrosis and active inflammation)<br>- Reflux esophagitis grade I, classification according to Savary and Miller<br>- former Smoker (Non-Smoker since 2006)<br>- Mother: pulmonary fibrosis, father COPD	- Total microorganism count: 100 germs/ml<br>- Culture result: *Candida albicans*	- Piperacillin 4.0 g/ Tazobactam 0.5 g i.v. about 1.5 hour before the operation<br>- Amoxicillin 875 mg / Clavulanic acid 125 mg operation day

**2**	- Chronic obstructive emphysema bronchitis (ID 2011)<br>- Long-term oxygen therapy (since 2017)<br>- Radiochemotherapy (up to 68 Gy and carboplatin/taxotere, Herceptin therapy) for breast cancer (ID 05/14)<br>- Mild arteriosclerosis in hypertension and hypercholesterolemia<br>- former Smoker 30py (Non-Smoker since 2011)	- Total germ count: >100.000 germs/ml<br>- culture result:<br>*S. aureus*	- Piperacillin 4 g/ Tazobactam 0.5 g i.v. about 1.5 hours before the operation
**3**	- Severe chronic obstructive emphysema bronchitis (ID 2007)<br>- Long-term oxygen therapy (since 2010)<br>- Non-invasive home ventilation because of sleep apnea syndrome (since 02/2020)<br>- Arterial hypertension<br>- Nicotine abuse (until 2008)<br>- Reflux esophagitis grade B, Los Angeles classification<br>- Lungs severely overinflated with a total lung capacity of 6.97l or 113%<br>- Respiratory global insufficiency according to capillary blood gas analysis	- Total germ count: >100.000 germs/ml<br>- culture result:<br>*S. aureus*,<br>*Candida albicans* (100 germs/ml), contamination by *throat flora* (500 germs/ml)	- Piperacillin 4 g/ Tazobactam 0.5 g i.v. about 45 min before the operation

Supplementary Table 2. Overview of previous diseases and therapies of the patients from elective lung operations.

Sample	Nicotine abuse	Respiratory diseases	Other relevant diseases	Antibiotics
L1	- 10 py<br>- Non-smoker for 16 years	House dust and hay fever allergy		Cefazolin 2 g on the day of the operation ~70 min before surgical procedure
L2	nk	Obstructive sleep apnea syndrome with CPAP mask since 04/13	- Diabetes mellitus (Type 2)<br>- Hypertension	Cefazolin 2 g on the day of the operation ~60 min before surgical procedure
L3	nk			Cefazolin 2g on the day of the operation ~75 min before surgical procedure
L4	- Smoker<br>- 40 py		- Hypertension	- none
L5	nk	- Chronic unilateral seropneumothorax basal (05/19)<br>- bound lungs with pleural fibrosis<br>- Intraoperatively destroyed lower lobe without gas exchange involvement recognizable *(location of sample!)*<br>- Covid-19 infection (12/2020)	- Diabetes mellitus (Type 2)<br>- Hypertension	- Cefuroxime 4x1.5 g up to 17 d before surgery<br>- i.v. Meropenem 3x1g from 16 d to the day of surgery for *P. aeruginosa* in total knee arthroplasty<br>- Cefazolin 2 g on the day of the operation ~25 min before the cut
L6	- Non-smoker		- Hypertension<br>- Radiation and chemotherapy (15/16) for prostate cancer (ID 09/15)<br>- Reflux esophagitis	- Cefazolin 2 g on the day of the operation ~15min before the cut
L7	nk	- Oropharyngeal carcinoma tonsil (ID 06/20)	- Radiation and chemotherapy cisplatin 5 cycles (08-09/20)	- Cefazolin 2 g on the day of the operation ~25 min before the cut
L8	- Non-smoker	- thoracoabdominal esophagectomy (09/20)<br>- Gastrointestinal stromal sarcoma esophagus (ID 09/20)<br>- COPD	- Adjuvant therapy with Imatinib 200mg since (11/20) with increasing nausea and vomiting lately	- Cefazolin 2 g on the day of the operation ~40 min before the cut

L9	nk	- Extended lower lobe resection right lung (06/19) because of<br>- Bronchial carcinoma (05/19) → *sample from upper lobe*	- Hypertension	- Cefazolin 2 g on the day of the operation ~40min before the cut
L10	nk	- Latent tuberculosis without an increased risk of reactivation	- Hypertension<br>- Amyotrophic Lateral Sclerosis (ALS)	- none
L11	nk	- During the operation, the lungs clearly adhered to the thoracic wall	- Hypertension	- Cefazolin 2 g on the day of the operation ~40min before the cut
L12	nk	- Esophageal varices<br>- Panlobular pulmonary emphysema<br>- Intraoperative: lungs with chronic dear altered and inflamed mucosa with cobblestone phenomenon in the sense of advanced COPD	- Liver transplantation (2016) because of Liver carcinom (ID 07/15)<br>- alcohol abuse	- Cefazolin 2 g on the day of the operation ~50min before the cut
L13	nk	- Tracheostomy tube with voice prosthesis<br>- Squamous cell carcinoma of the floor of the mouth anterior bilateral (ID 2020)<br>- Implementation of an attachment and placement osteoplasty in the lower jaw (03/21)<br>- Tongue solution and mouth floor plastic (09/20)<br>- Laryngeal carcinoma (ID 04/2021)<br>- laryngectomy (05/21)<br>- Hypopharyngeal stenosis	- adjuvant IMRT radiation (06-08/22)	- Cefazolin 2 g on the day of the operation ~40 min before the cut
L14	nk	- Bronchial asthma	- Radiation 06/2020 (chest/chest wall right: GD 40.05 Gy) because of breast cancer (ID 01/20)	- Clindamycin 600 mg on the day of the operation ~30 min before the cut
L15	nk	- Mild restrictive ventilation disorder because of<br>- Left upper lobe lobectomy because of adenocarcinoma (03/2014) (→ *sample from right lobe)*	- latent Typ 2 diabetes mellitus)<br>- Arterielle Hypertension<br>- adjuvant chemotherapy with Capecitabine (2014)	- Cefazolin 2 g on the day of the operation ~35 min before the cut

		- tonsillectomy		
L16	nk		- Typ 2 Diabetes mellitus)<br>- Hypertension	- Cefazolin 2 g on the day of the operation ~50 min before the cut
L17	nk	- Squamous cell carcinoma esophagus (2 months prior Sample ID)		- none
L18	- Non- smoker			- Cefazolin 2 g on the day of the operation ~10 min before the cut
L19	nk	- SARS-CoV-2 positive March 22 and Dec 21	- reflux esophagitis	- Piperacillin 4 g/ Tazobactam 0.5 g i.v. for ~1 w, ~ 3 w before operation<br>- Amoxicillin 1000 mg/Clarythromycin 500 mg 1-0-1 for 10 d to 6 d before sample collection<br>- Cefazolin 2 g on the day of the operation ~80min before the cut

nk- not known, ID- Initial diagnosis

Supplementary Table 3. Read count of operational taxonomic units (OTUs) across all samples. The column "is_contaminant" indicates whether the OTU is classified as a contaminant.
